# Level and relationships of academic skills and family functions with academic performance during Covid 19 pandemic

**DOI:** 10.1186/s41983-022-00592-5

**Published:** 2022-12-05

**Authors:** Muath A. Alammar, Dushad Ram, Ibrahim Abdulrahman Almansour, Abdulrhman Saad Aljammaz

**Affiliations:** 1 Department of Medicine (Family Medicine), Shaqra College of Medicine, Shaqra University, Shaqra, Saudi Arabia; 2 Department of Medicine (Psychiatry), Shaqra College of Medicine, Shaqra University, Shaqra, 15526-7487 Saudi Arabia; 3 College of Medicine, Shaqra University, Shaqra, Saudi Arabia

**Keywords:** Academic skills, Family function, Academic performance, College students

## Abstract

**Background:**

The research indicates that academic skills and family function may influence academic achievement. The Covid 19 epidemic has impacted regular academic and family function. However, there is a dearth of studies evaluating the relevance of academic skills and family function on college students' academic achievement. This research was done to determine the levels and relationships between academic achievement and study skills and family functions. Two hundred seventy-nine college students were assessed with Sociodemographic and academic proforma, Study Skills Assessment Questionnaire (SSAQ), and The McMaster Family Functioning Scale (MFFS).

**Results:**

Results revealed that the mean score of SSAQ and MFFS were 179.92 and 17.88, respectively. Multiple regression analysis showed that the score of reading skills and the MFFS score statistically significantly predicted the score of the exam.

**Conclusions:**

On the basis of this study's results, it is possible to conclude that reading skills and family functioning may influence academic success.

## Background

Academic performance is usually the most important element in deciding whether or not to offer a university degree. One of the most important markers of a student's academic performance is their Grade Point Average (GPA). A grade point average (GPA) is a numerically weighted average of a student's grades for the course of his studies that can be used to assess a student's academic performance and position. Students' academic performance may be influenced by internal factors such as study skills and their immediate environment, such as family, as the Covid-19 epidemic impacted a significant proportion of the Arabic population [1]**.** Academic performance may be impacted by poor sleep quality, somatization, depression, anxiety, post-traumatic stress disorder, and psychosis after infection [2, 3]. Similarly, withdrawal, anxiety/depression, physical difficulties, internalizing problems, and externalizing problems may have impacted the academic performance of younger age groups [4]. Infection or death of family members as a result of Covid-19; social and parental issues; increased family expenses; and a decrease in family income indirectly affect academic achievement [4]. During the Covid 19 outbreak, there were also considerable levels of fear and stigmatization [5, 6]. Thus, it is plausible that the COVID-19 outbreak may have directly or indirectly affected students' academic performance. Aside from this, the Covid 19 outbreak altered teaching and learning, with students studying online from sitting at home. The Covid 19 epidemic has also affected family functioning, which may impact students' learning environments [7]. Thus, there was a need to study the academic and family variables among college students during the pandemic.

Study skills are an array of skills that requires training and practice with specific techniques that help a learner acquire, organize, retain, and use information [8]. Study skills include the competencies associated with acquiring, recording, organizing, synthesizing, remembering, and using information [9]. Academic competence is associated with an understanding of and ability to apply effective study skills [9]. Study skills play an important role in university students in academic performance [10, 11]. However, such a role was under-explored during the Covid 19 pandemic, when the online mode of academic activities was predominant. Multiple factors appear to influence the study skills, such as school and society. Indirect evidence suggests that family may also be an important determinant [12]. Higher academic performance was reported to be associated with more parental support and involvement in academic activities [13]. Also, that general social support positively affects general academic achievement [14, 15]. Indirect evidence suggests that family and social factors influence study skills among Saudi students [16]. While it appears that the relationship between academic performance and family function is beneficial during the non-pandemic period, it has yet to be established during the pandemic among Saudi students.

## Methods

After obtaining approval from the institutional ethics committee, this cross-sectional study was conducted at Shaqra University in Saudi Arabia. An online Google form was created to collect data, and a link to a WhatsApp group of students from various colleges at—University was provided. The form did not include any items that could be used to identify the respondent, and there was an option to opt-out of the study.

The research began on April 12, 2021, and ended on August 31, 2021. The study included participants of any gender who were members of Shaqra University's colleges and had completed a 6-month course. Individuals with a broken family or who live separately were excluded. By the deadline, 279 responses had been received from individuals who met the study's inclusion criteria. The assessment tools were:

1. Sociodemographic and academic proforma: It consisted of age, gender, college of affiliation, level, GPA score, experiencing any difficulty in the study, difficulty in understanding teaching, perceived course suitability, and perceived favorable family environment. Prior semester exam score was GPA that ranged from 1 to 5 and was obtained by dividing the course credit hours by course point.
2. Study Skills Assessment Questionnaire (SSAQ): The tool assesses eight domains: time management and procrastination, concentration and memory, study aids and note-taking, test strategies and test anxiety, organizing and processing information, motivation, and attitude, reading and selecting the main idea, and writing [7]. Eight items were used to assess each domain, each of which was scored on a five-point Likert scale (always, often, sometimes, rarely, never), and its score range was from 5 to 1. The minimum and maximum scores for each domain are 6 and 30. A score of less than 50% in each domain indicated poor study skills; a score of 50% to 75% in each domain indicated moderate study skills; a score of more than 75% in each domain indicated good study skills. The scale is in use in Saudi Arabia [17].
3. The McMaster Family Functioning Scale (MFFS): This scale assesses the degree to which a family has constructive and supportive relationships [18]. This 12-item additive scale provides a global assessment of family functioning by including indicators for problem-solving, communication, affective involvement, affective responsiveness, conflict resolution, and behavior control. Odd items are Score in reverse order. A participant's family functioning score is the sum of the 12 items, ranging from 12 to 48, with higher scores indicating worse family functioning. Scale is being used in Saudi Arabia in the Arabic version [19].

IBM SPSS version 25.0 was used to analyze the data. The demographic, study skills and family function variables were analyzed using descriptive statistics. Independent Student’s T-tests were used to determine the relationships between study skills and family function with demographic and academic variables. Multiple regression analysis was used to determine whether the value of the SSAQ and MFFS scores can predict the value of the exam score. The significance of statistical analysis was 0.05.

## Results

Sociodemographic features characterized by the majority being female, single, from health science, did not report major difficulty in the study, satisfied with the course they were perusing, the family was supportive for their academic course and good study skills (Table 1).

**Table 1:** Sociodemographic features

	Frequency	Percent
Gender		
Male	131	47.0
Female	148	53.0
Social status		
Single	266	95.3
Married	13	4.7
College		
Health sciences	142	50.9
Non-health sciences	137	49.1
Difficulty in study		
Yes	28	10.0
No	251	90.0
Difficulty in understanding teaching		
Yes	85	30.5
No	194	69.5
Perceived course suitability		
Yes	254	91.0
No	25	9.0
Supportive family environment		
Yes	262	93.9
No	17	6.1
Study skills		
Mild	36	12.9
Moderate	82	29.4
Good	160	57.3

The mean GPA, MFFS, and SSAQ scores were 4.24, 17.88, and 179.92, respectively. The student has a better score on SSAQ subscale Concentration/Memory (23.58), Readiness to take an exam (23.65), Attitude/Motivation (23.42); while lesser in Time management (22.08), Aid/Notetaking (22.03), Organizing information (22.57), Reading skill (21.89); and lowest in Writing skill (20.67) (Table 2).

**Table 2:** Demographic and academic features

Variables	Mean	Std. deviation
Age	21.23	2.67
Exam score (GPA)	4.24	0.59
SSAQ Time management	22.08	6.94
SSAQ Concentration and Memory	23.58	6.77
SSAQ Notetaking	22.03	7.35
SSAQ Readiness to take an exam	23.65	6.89
SSAQ Organizing information	22.57	7.28
SSAQ Attitude/Motivation	23.42	7.09
SSAQ Reading skill	21.89	7.43
SSAQ Writing skill	20.67	6.63
SSAQ Total score	179.92	51.67
MFFS Score	17.88	8.37

*SSAQ* Study Skills Assessment Questionnaire; *MFFS* McMaster Family Function Scale

There was an statistically significant group difference by difficulty in understanding on score of SSAQ subscale Time management (*t* = − 4.15, *df* = 277, *p* = 0.001), Note taking (*t* = − 3.59, *df* = 277, *p* = 0.001), Readiness to take an exam (*t* = − 2.37, *df* = 277, *p* = 0.018), Organizing information (*t* = − 3.78, *df* = 277, *p* = 0.001), Attitude Motivation (*t* = − 2.40, *df* = 227, *p* = 0.017), main idea/self testing (*t* = − 3.12, *df* = 277, *p* = 0.002), and Writing skill (*t* = − 4.32, *df* = 277, *p* = 0.001) (Table 3). Also, there was a statistically significant group difference on score of SSAQ subscale Writing skill by gender (*t* = − 2.45, *df* = 277, *p* = 0.015) and Attitude/Motivation by supportive family environment (*t* = 2.42, *df* = 277, *p* = 0.016) (Table 3).

**Table 3:** Relationships of study skill and family function with demographic and academic variables

	*t*-test for equality of means						
	*t*	*df*	Sig. (2-tailed)	Mean difference	Std. error difference	95% confidence interval of the difference	
						Lower	Upper
SSAQ Writing skill*Gender	− 2.45	277	0.015	− 1.93	0.78	− 3.49	− 0.38
SSAQ Time management*difficulty in understanding teaching	− 4.15	277	0.001	− 3.64	0.87	− 5.37	− 1.91
SSAQ Note taking*difficulty in understanding teaching	− 3.59	277	0.001	− 3.36	0.93	− 5.20	− 1.51
SSAQ Readiness to take an exam*difficulty in understanding teaching	− 2.37	277	0.018	− 2.11	0.88	− 3.86	− 0.35
SSAQ Organizing information*difficulty in understanding teaching	− 3.78	277	0.001	− 3.50	0.92	− 5.32	− 1.68
SSAQ Attitude Motivation*difficulty in understanding teaching	− 2.40	277	0.017	− 2.19	0.91	− 4.00	− 0.39
SSAQ Reading skill*difficulty in understanding teaching	− 3.12	277	0.002	− 2.97	0.95	− 4.85	− 1.10
SSAQ Writing skill*difficulty in understanding teaching	− 4.32	277	0.001	− 3.62	0.83	− 5.26	− 1.97
SSAQ Attitude/Motivation* Supportive family environment	2.42	277	0.016	4.27	1.76	0.80	7.73
MFFS Score*Gender	− 3.59	277	0.001	− 3.53	0.98	− 5.47	− 1.60
MFFS Score*difficulty in Study	2.79	277	0.005	4.61	1.64	1.36	7.85
MFFS Score*Perceived course suitability	− 5.12	277	0.001	− 8.60	1.68	− 11.91	5.29
MFFS Score* Supportive family environment	− 3.44	277	0.001	− 7.07	2.05	− 11.12	− 3.02

*SSAQ* Study Skills Assessment Questionnaire; *MFFS* McMaster Family Function Scale

On the score of MFFS there was a statistically significant group difference by gender (*t* = − 3.59, *df* = 277, *p* = 0.001), difficulty in Study (*t* = 2.79, *df* = 277, *p* = 0.005), Perceived course suitability (*t* = − 5.12, *df* = 277, *p* = 0.001) and Supportive family environment (*t* = − 3.44, *df* = 277, *p* = 0.001) (Table 3).

Multiple linear regression analysis (Adjusted *R* = 0.047, df-11, *F* = 2.224, *p* = 0.014) was done using the SSAQ subscale score and MFFS score as the predictor variable and exam score as the dependent variable. Value on SSAQ subscale Reading skill score statistically significantly negatively predicted the value on the Score of exam score (*p* = 0.018). Similarly, the value of the MFFS Score statistically significantly negatively predicted the value of the Score of exam score (*p* = 0.003) (Table 4).

**Table 4:** Relationships of demographic, academic variables with exam score

	Model	Unstandardized coefficients		Standardized coefficients	*t*	Sig
		*B*	Std. Error	Beta		
1	(Constant)	4.976	0.365		13.61	0.001
	Age	− 0.031	0.016	− 0.142	− 1.91	0.057
	Level	0.017	0.016	0.082	1.08	0.278
	SSAQ Time management	0.011	0.013	0.128	0.86	0.386
	SSAQ Concentration and memory	0.000	0.014	0.005	0.02	0.977
	SSAQ Aid/Notetaking	0.009	0.012	0.113	0.77	0.437
	SSAQ Readiness to take an exam	0.007	0.014	0.078	0.47	0.632
	SSAQ Organizing information	0.001	0.012	0.015	0.10	0.918
	SSAQ Attitude/Motivation	0.006	0.013	0.074	0.49	0.624
	SSAQ Reading skill	− 0.027	0.011	− 0.340	− 2.37	0.018
	SSAQ Writing skill	− 0.005	0.010	− 0.054	− 0.50	0.613
	MFFS Score	− 0.013	0.005	− 0.191	− 2.96	0.003

Dependent Variable: Exam Score. Model summary: Adjusted *R* = 0.047, *df*-11, *F* = 2.224, *p* = 0.014

*SSAQ* Study Skills Assessment Questionnaire; *MFFS* McMaster Family Function Scale

## Discussion

The study examined the relationship between academic performance, study skills, and family functioning during the Covid 19 pandemic. There is a paucity of studies reporting these variables in Arabic countries to compare the findings. The academic skills score obtained in this study was comparable to another study [16]. They observed, however, that the majority of students possessed a mild to moderate level of academic skills, whereas the majority in this study possessed good skills. Another report from the same geographic region indicates a lower level of academic skills across multiple domains, which contrasts with this study [20]. This difference could be explained by the fact that dentistry courses enroll a greater number of students than other courses in their study sample and have no influence on the pandemic in academic activities. The mean MFFS Score was lower than in another Chinese report before the pandemic [21].

A significant finding of this study was the presence of a significant relationship between difficulty in comprehending the teaching and the majority of the study skills subscale score. A meta-analysis reveals that motivation and social support aid in academic performance and learning [22]. According to intervention studies, study skills improve one's comprehension of a subject and thus one's ability to learn [20, 23]. The type of college and course have an effect on the levels of academic subscale scores [24]. Family support enables students to develop stronger learning skills and cope with academic difficulties [25].

Another significant finding of this study was the occurrence of a significant relation between exam scores with reading skills and family function. Similar to this study's findings, an earlier report from South Africa indicates that reading skills and habits are associated with academic performance among undergraduate students [26, 27]. Earlier research also indicates that developing academic skills contributes to academic achievement [28, 29]. This study found a positive link between academic performance and better family function. Family is an important factor linked to academic performance [30]. Family conflicts have a detrimental effect on students' academic performance [31]. Joint decision-making within the family has been associated with improved academic performance, while greater disagreement with family members has been associated with poor performance [32]. The family appears to have an effect on academic performance via academic self-efficacy or students' perception of progress toward academic goals [22]. The more favorable the family's intellectual climate, the more mature students' beliefs about learning are, resulting in deep and metacognitive strategies and academic performance [33]. According to reports, family functioning and social support boost self-esteem, which in turn influences academic performance [34].

## Conclusions

As a result of the findings, it may be concluded that family function might﻿ influences the academic performance. The findings imply that family function should be examined when college students' academic performance is impaired, and that family members should be educated about the family's role in academic performance. This conclusion must be confirmed through additional research.

## Limitations

The study's findings should be interpreted in the background of the study's limitations, including a cross-sectional online study that relied on snowball sampling and could not determine the response rate, and there was no control group. Future studies should address these issues along with the other determinant of family function and academic skills and performance.

## Data Availability

The datasets used and/or analyzed during the current study are available from the corresponding author upon reasonable request.

## Abbreviations

GPA: Grade Point Average
SSAQ: Study Skills Assessment Questionnaire
MFFS: The McMaster Family Functioning Scale

## References

[1] Alkhamees AA, Alrashed SA, Alzunaydi AA, Almohimeed AS, Aljohani MS (2020). The psychological impact of COVID-19 pandemic on the general population of Saudi Arabia. Compr Psychiatry.

[2] Alkathiri MA, Almohammed OA, Alqahtani F, AlRuthia Y (2022). Associations of depression and anxiety with stigma in a sample of patients in Saudi Arabia who recovered from COVID-19. Psychol Res Behav Manag.

[3] Ahmed GK, Khedr EM, Hamad DA, Meshref TS, Hashem MM, Aly MM (2021). Long term impact of Covid-19 infection on sleep and mental health: a cross-sectional study. Psychiatry Res.

[4] Ahmed GK, Elbeh K, Gomaa HM, Soliman S (2021). Does COVID-19 infection have an impact on children's psychological problems?. Middle East Current Psychiatry.

[5] Almoayad F, Mahboub S, Amer LB, Alrabiah A, Alhashem A (2021). Stigmatisation of COVID-19 in Riyadh, Saudi Arabia: a cross-sectional study. Sultan Qaboos Univ Med J.

[6] Osman DM, Khalaf FR, Ahmed GK, Abdelbadee AY, Abbas AM, Mohammed HM (2022). Worry from contracting COVID-19 infection and its stigma among Egyptian health care providers. J Egypt Public Health Assoc.

[7] Study Skills Assessment Questionnaire | University of Houston-Clear Lake. Accessed September 4, 2021. https://uhcl.edu/counseling-services/resources/study-skills-questionnaire.

[8] Rohwer WD (1984). An invitation to an educational psychology of studying. Educational Psychologist.

[9] Gettinger M, Seibert JK (2002). Contributions of study skills to academic competence. Sch Psychol Rev.

[10] Hassanbeigi A, Askari J, Nakhjavani M (2011). The relationship between study skills and academic performance of university students. Procedia Soc Behav Sci.

[11] Shetty SS, Srinivasan SR (2014). Effectiveness of study skills on academic performance of dental students. J Educ Ethics Dentistry.

[12] Grafton-Clarke C, Garner J (2018). Study skills, social life and confidence in undergraduate medical students. MedEdPublish..

[13] Shahzad M, Abdullah F, Fatima S, Riaz F, Mehmood S (2020). Impacts of parental support on academic performance among secondary school students in Islamabad. J Soc Sci.

[14] Fass ME, Tubman JG (2002). The influence of parental and peer attachment on college students' academic achievement. Psychol Sch.

[15] Silva AD, Vautero J, Usssene C (2021). The influence of family on academic performance of Mozambican university students. Int J Educ Dev.

[16] Rab SDA, Islam M, Alrefaai I. The General Study Habits of Major EFL. Students in King Khalid University and their Relationships With GPA, Gender and. Certain Social Factors. *undefined*. Published online 2013. Accessed September 4, 2021. https://www.semanticscholar.org/paper/The-General-Study-Habits-of-Major-EFL.-Students-in-Rab-Islam/8d6f5c4b0992e640d5b1fa18aa0b1ae7d56e178a.

[17] Kamel Ashraf MF, Behery F, Kenawy G (2020). Exploring study skills among university students in Riyadh, Saudi Arabia. Saudi J Oral Sci..

[18] Miller IW, Epstein NB, Bishop DS, Keitner GI (1985). The McMaster family assessment device: reliability and validity. J Marital Fam Ther.

[19] Hatw MME, Taher AAE, Hamidi AE, Alturkait FA (2015). The association of exposure to the 2009 south war with the physical, psychological, and family well-being of Saudi children. SMJ.

[20] Hedin B, Kann V (2019). Improving study skills by combining a study skill module and repeated reflection seminars. Educ Res Int.

[21] Chen J, Xu D, Wu X (2019). Seeking help for mental health problems in Hong Kong: the role of family. Adm Policy Ment Health.

[22] Robbins SB, Lauver K, Le H, Davis D, Langley R, Carlstrom A (2004). Do psychosocial and study skill factors predict college outcomes? A meta-analysis. Psychol Bull.

[23] Alexander DF (1985). The effect of study skill training on learning disabled students' Re℡ling of expository material. J Appl Behav Anal.

[24] Dwarika-Bhagat N, Sa B, Majumder AA (2017). Does study skill matter? A descriptive study on undergraduate health profession students in the University of the West Indies. Educ Med J.

[25] Cutrona CE, Cole V, Colangelo N, Assouline SG, Russell DW (1994). Perceived parental social support and academic achievement: an attachment theory perspective. J Pers Soc Psychol.

[26] Owusu-Acheaw M. Reading Habits Among Students and its Effect on Academic Performance: A Study of Students of Koforidua Polytechnic. *undefined*. Published online 2014. Accessed September 3, 2021. https://www.semanticscholar.org/paper/Reading-Habits-Among-Students-and-its-Effect-on-A-Owusu-Acheaw/14c8f8bba72a970705c71747c75d6bf124cc8432.

[27] Pretorius EJ (2000). Reading and the Unisa student: is academic performance related to reading ability?. Progressio.

[28] Cox SR, Friesner DL, Khayum M (2003). Do reading skills courses help underprepared readers achieve academic success in college?. J College Read Learn.

[29] Gao Q, Wang H, Mo D, Shi Y, Kenny K, Rozelle S (2018). Can reading programs improve reading skills and academic performance in rural China?. China Econ Rev.

[30] Walker KL, Satterwhite T (2002). Academic performance among African American and Caucasian college students: is the family still important?. Coll Stud J.

[31] Bahrassa NF, Syed M, Su J, Lee RM (2011). Family conflict and academic performance of first-year Asian American undergraduates. Cultur Divers Ethnic Minor Psychol.

[32] Dornbusch SM, Ritter PL, Mont-Reynaud R, Chen Z (1990). Family decision making and academic performance in a diverse high school population. J Adolesc Res.

[33] Cano F, Cardelle-Elawar M, Khine MS (2008). Family environment, epistemological beliefs, learning strategies, and academic performance: a path analysis. Knowing, knowledge and beliefs: epistemological studies across diverse cultures.

[34] Shi J, Wang L, Yao Y, Su N, Zhao X, Zhan C (2017). Family function and self-esteem among Chinese University Students with and without grandparenting experience: moderating effect of social support. Front Psychol.

